# Successful Long‐Term Survival With Various Modalities of Treatments Combining Minimally Invasive Surgery for Intrahepatic and Extrahepatic Recurrence of Hepatocellular Carcinoma: A Case Report

**DOI:** 10.1155/cris/2481885

**Published:** 2026-08-07

**Authors:** Kit-Fai Lee, Stephen Lam Chan, Leung Li, Cheuk-Man Chu, Charing C. N. Chong, John Wong, Kenneth S. H. Chok

**Affiliations:** ^1^ Department of Surgery, Prince of Wales Hospital, The Chinese University of Hong Kong, Hong Kong, China, cuhk.edu.hk; ^2^ Department of Clinical Oncology, Prince of Wales Hospital, The Chinese University of Hong Kong, Hong Kong, China, cuhk.edu.hk; ^3^ Department of Imaging and Interventional Radiology, Prince of Wales Hospital, The Chinese University of Hong Kong, Hong Kong, China, cuhk.edu.hk

**Keywords:** extrahepatic recurrence, hepatocellular carcinoma, intrahepatic recurrence, minimally invasive surgery

## Abstract

A 63‐year‐old man received a robotic left lateral sectionectomy for a 2 cm solitary segment (seg) 2/3 hepatocellular carcinoma (HCC). Pathology revealed clear margins without vascular invasion. Three years later patient received transarterial chemo‐embolization (TACE) for suspected intrahepatic recurrence. However, follow‐up computed tomography (CT) revealed solitary peritoneal metastasis which increased to 8 cm at splenic hilum despite targeted therapy and immunotherapy was given, thus a robotic excision of the peritoneal metastasis with splenectomy was performed. CT at 3 months after surgery revealed solely new intrahepatic recurrences at segs 6 and 7. A total of four TACE were given subsequently. Laparoscopic seg 6/7 wedge liver resection was performed for residual disease. Two further small recurrences occurred at seg 7 at 2 years after surgery; stereotactic body radiotherapy (SBRT) was given. Ultimately, the patient was disease‐free and enjoyed good quality of life for more than 10 years since the initial operation despite episodes of extrahepatic and intrahepatic recurrences. Thus, a multidisciplinary approach for HCC management is important to optimize treatment outcomes. Long‐term cure is possible with multimodality treatment for recurrent HCC, and the use of minimally invasive surgery can enhance patient’s quality of life.

## 1. Introduction

Hepatocellular carcinoma (HCC) is the sixth most common cancer around the world [1]. Hepatectomy remains the mainstay curative treatment for the disease [2]. However, recurrence, even after radical resection, is common. The recurrence usually occurs within the liver, while extrahepatic recurrence carries a much worse prognosis [3].

Curative treatments for intrahepatic recurrent diseases include repeat hepatectomy and local ablation [4]. Salvage liver transplantation is indicated in selected patients [5]. The management of extrahepatic recurrence is more complicated, and a multidisciplinary approach is desirable. Treatment usually consists of systemic treatment with resection of oligo‐metastasis in selected patients [6].

Here, we would like to report a patient with recurrent HCC, both intrahepatic and extrahepatic, for which surgical resection, targeted therapy, immunotherapy, transarterial chemo‐embolization (TACE), and stereotactic body radiotherapy (SBRT) were given at different stages of disease. Ultimately, the patient was disease‐free and enjoyed a good quality of life for more than 10 years after initial surgery.

The present case is unique in that long‐term survival can be achieved with various surgical and nonsurgical interventions despite repeated recurrent extrahepatic and intrahepatic HCC. This underscores the importance of involvement of different specialties in the form of a multidisclipinary approach for management of HCC. Furthermore, the adoption of a minimally invasive surgical approach and liver parenchyma‐preserving resection not only improves short‐term surgical outcomes but also makes subsequent retreatment more feasible [7, 8].

## 2. Case Presentation

**Figure 1:**
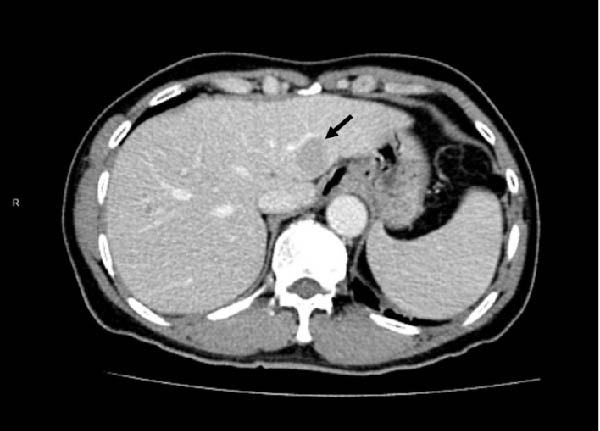
CT showing a segment 2/3 tumor (arrow).

A 63‐year‐old man who had a history of treated pulmonary tuberculosis and renal impairment was diagnosed to have a small solitary segment (seg) 2/3 HCC in December 2013 (Figure 1). He was a hepatitis B carrier on entecavir. His liver function was normal, and alpha‐fetoprotein (AFP) was 2 µg/L only. Robotic left lateral sectionectomy was performed in January 2014. The operation was uneventful. The pathology confirmed a 2 cm moderately differentiated HCC with a 1.5 cm resection margin without vascular invasion.

**Figure 2:**
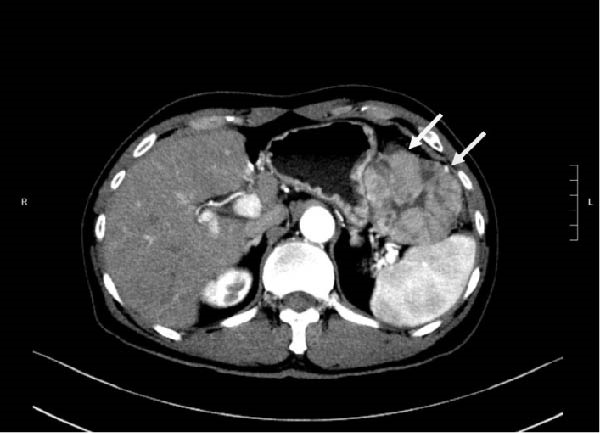
CT showing a peritoneal recurrent tumor near the splenic hilum (arrows).

Serial follow‐up computed tomography (CT) in July 2014 and January 2015 revealed no recurrent disease. However, the AFP was found to be elevated to 118 µg/L in February 2017. Dual tracer positron emission tomography (PET) CT with C^11^ acetate and FDG in March 2017 revealed a few small arterial enhancing nodules in the liver, the largest 6 mm at seg 4a/b. In view of suspected multifocal small recurrent HCC, TACE was given two times, with the last one in October 2017. However, the AFP continued to rise to 3603 µg/L despite TACE. Repeated dual tracer (FDG and C‐acetate) PET‐CT in November 2017 revealed a 2.7 cm peritoneal recurrence close to the splenic hilum without intrahepatic recurrence. The patient was given sorafenib from December 2017 to March 2018. A follow‐up CT in March 2018 showed that the peritoneal metastasis increased to 5.2 cm. He was recruited to a clinical trial and given anti‐PD1‐immunotherapy. On repeated PET CT in October 2018, the solitary peritoneal metastasis further increased to 8 cm at the splenic hilum (Figure 2) and AFP further increased to 5105 µg/L.

Robotic excision of the peritoneal metastasis with splenectomy was performed in Nov 2018. A friable intraperitoneal tumor adherent to the splenic hilum was found. The pathology confirmed a 10 cm HCC with the margin focally involved by the tumor. There was focal lymphovascular permeation; otherwise, the microscopic features were similar to the primary tumor. The spleen was not involved. The AFP decreased to 2 µg/L in January 2019 after surgery.

**Figure 3:**
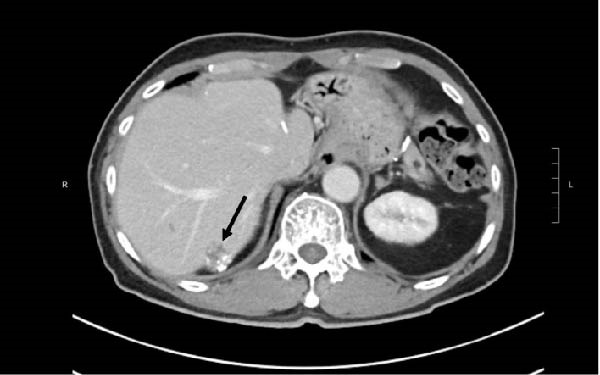
CT showing a recurrent seg 6/7 tumor (arrow).

However, another CT in Feb 2019 revealed new intrahepatic recurrences at seg 7 (1.8 and 1.1 cm) and seg 6/7 (1.1 cm). TACE was given in April 2019 and September 2019. Since viable tumors still presented on CT in December 2019, two more TACEs were given, the last one in December 2020. Follow‐up CT in April 2021 revealed a residual lesion at seg 6/7 (Figure 3). In view of limited residual disease, laparoscopic seg 6/7 wedge liver resection was performed in June 2021. The pathology revealed a 2.4 cm moderately differentiated HCC with a minimal resection margin of 1 mm. The microscopic features were similar to the primary tumor, except there was microvascular invasion.

**Figure 4:**
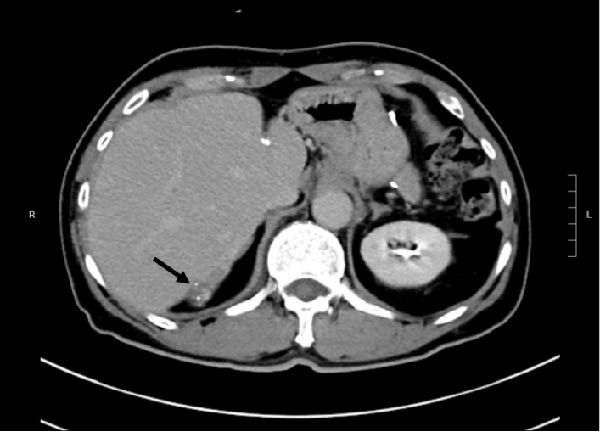
CT showing a small recurrent tumor at seg 7 (arrow).

**Figure 5:**
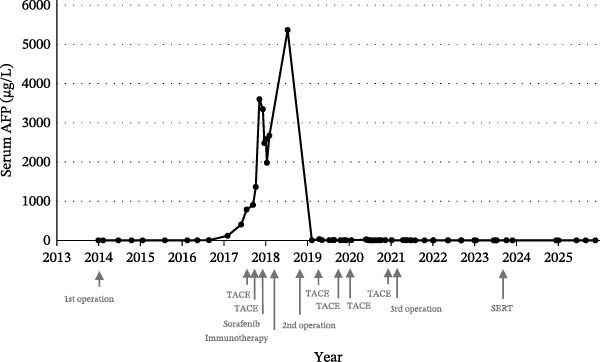
Graph showing the serial change of serum alpha‐fetoprotein (AFP) level with various interventions over time. 1st operation: robotic left lateral sectionectomy. 2nd operation: robotic excision of peritoneal metastasis with splenectomy. 3rd operation: laparoscopic segment 6/7 wedge resection. SBRT, stereotactic body radiotherapy; TACE, transarterial chemo‐embolization.

CT in June 2023 revealed two small recurrences at seg 7 (Figure 4), SBRT was given. Follow‐up CT in February 2024 and in December 2024 revealed no further recurrence. The patient’s latest AFP was 2 µg/L, and his liver function was normal in January 2025. The serial change of serum AFP level with various interventions over time is shown in Figure 5. Table 1 summarizes the tumor sites, treatment modalities, pathological findings, and treatment outcomes for this patient. All along, the patient remained well with minimal symptoms despite multiple various treatments he has received.

**Table 1:** The tumor sites, treatment modalities, pathological findings, and treatment outcomes of the patient.

Tumor site	Treatment	Pathological finding	Outcome
Seg 2/3	Resection	2 cm HCC	No recurrence
Peritoneal metastasis close to splenic hilum	TACE		No response
	Sorafenib		No response
	Immunotherapy		No response
	Resection	10 cm HCC	No recurrence
Segs 6/7 and 7	TACE		Residual tumor at seg 6/7
	Resection	2.4 cm HCC	No recurrence
Seg 7	SBRT		No recurrence

Abbreviations: SBRT, stereotactic body radiotherapy; TACE, transarterial chemo‐embolization.

## 3. Discussion

Our patient initially presented with a small HCC for which surgical resection was the best optimal treatment for him. The location of the tumor in the left lateral section of the liver also represented an ideal location for a minimally invasive approach [9]. Local ablation is an alternative treatment for small HCC but has higher recurrence and lower overall survival compared with laparoscopic hepatectomy [10]. The first recurrent disease was revealed by the rapid increase in AFP around 3 years after the operation. The failure of the first two treatments of TACE was likely due to misdiagnosed intrahepatic recurrence; instead, the recurrence occurred outside the liver. This was a solitary peritoneal metastasis near the splenic hilum. In view of the general poor prognosis due to the spread of disease outside the liver, systemic treatment with targeted therapy and then immunotherapy was given as these were recommended treatments for extrahepatic recurrent HCC [11–14]. However, the tumor increased rapidly in size, and AFP further increased despite these treatments, and at the same time, there was no evidence of other extrahepatic or intrahepatic disease on follow‐up scans, so resection of the solitary peritoneal metastasis was offered to the patient [15]. It was done again with a minimally invasive approach as it was feasible with superior short‐term outcomes than the open approach. Though the resection margin was involved, the AFP returned to normal afterward, and there was no further extrahepatic recurrence noted. Cytoreductive surgery with hyperthermic intraperitoneal chemotherapy has been tried for HCC peritoneal metastasis with favorable results in selected patients [16]. This treatment was more indicated for diffuse peritoneal metastases rather than for a large solitary metastasis as in this case. Retrospectively, metastectomy could be considered earlier as the peritoneal metastasis was solitary without evidence of concurrent intrahepatic or other extrahepatic recurrence.

The exact cause of the occurrence of the solitary peritoneal recurrence is uncertain. Tumor seeding to the peritoneum during laparoscopic hepatectomy is one postulation, but it is unlikely as the surgery was smooth without breaching of the tumor capsule and the resection margin was more than adequate (1.5 cm). The long interval of 3 years for the disease to recur also makes this hypothesis less likely. Another interesting observation is that the recurrent tumor was associated with high AFP, while the initial tumor was associated with a normal AFP level. Such a phenomenon has been reported in a study with an incidence of 28 out of 400 patients (7%) [17]. Thus, the two tumors may represent different cell lines. Such divergence underscores the inherent heterogeneity prevalent in HCC [18]. Unfortunately, no molecular or genetic investigation was performed on resected specimens. Apparently, the peritoneal recurrence probably represented a biologically distinct tumor, as it was associated with high AFP and responded well to resection, while the multiple episodes of intrahepatic recurrence with normal AFP represented a more treatment‐resistant tumor. However, why the second tumor occured in the peritoneum still cannot be explained.

The subsequent occurrence of small intrahepatic recurrence was managed with TACE and with laparoscopic wedge resection for residual disease after TACE. Local ablation therapy was not used in our patient as the lesions were not feasible for a percutaneous approach, while laparoscopic resection would be better than laparoscopic ablation for treatment of subcapsular lesions. Lastly, SBRT was able to manage the small recurrences without subject the patient to open surgery. Further TACE was not considered due to the worry of worsening liver function, as the patient had four TACE treatments before. Though the patient became disease‐free at the end, several treatment modalities were needed for disease control. Some treatment failures could be due to mismatched disease characteristics. Additional pathological assessment and molecular characterization could be beneficial in guiding the optimal treatment option targeting the dominant disease.

Thus, despite the multiple various treatments for the recurrent HCC he has received, our patient could still enjoy the benefit of minimally invasive surgery with good quality of life and preserved liver function. Similar long‐term survival has been reported in other malignant tumors like solitary fibrous tumor managed with multiple various treatments including surgical resection, radiotherapy, chemotherapy, and targeted therapy [19]. Thus, it is crucial not to abandon treatment for malignant tumors if treatment options are available.

## Funding

No funding was received for this study.

## Ethics Statement

This study was performed in line with the principles of the Declaration of Helsinki.

## Consent

Written informed consent was obtained from the patient for the publication of this case report and its accompanying images. A copy of the consent form is available for review by the Editor‐in‐Chief upon request.

## Conflicts of Interest

The authors declare no conflicts of interest.

## Data Availability

The data that support the findings of this study are available upon request from the corresponding author. The data are not publicly available due to privacy or ethical restrictions.
